# Supplementary material for: Otolith chemoscape analysis in whiting links fishing grounds to nursery areas
Source: Commun Biol. 2020 Nov 19;3:690. doi: 10.1038/s42003-020-01433-y (PMC7677557; doi:10.1038/s42003-020-01433-y)
Supplement: Supplementary file 2 — Reporting Summary [file 42003_2020_1433_MOESM2_ESM.pdf]

nature research

Corresponding author(s): Neil M. Burns - neil.burns@sruc.ac.uk

Last updated by author(s): Oct 9, 2020

Reporting Summary

Nature Research wishes to improve the reproducibility of the work that we publish. This form provides structure for consistency and transparency in reporting. For further information on Nature Research policies, see our Editorial Policies and the Editorial Policy Checklist.

Statistics

For all statistical analyses, confirm that the following items are present in the figure legend, table legend, main text, or Methods section.

n/a	Confirmed	
☐	☒	The exact sample size (*n*) for each experimental group/condition, given as a discrete number and unit of measurement
☐	☒	A statement on whether measurements were taken from distinct samples or whether the same sample was measured repeatedly
☐	☒	The statistical test(s) used AND whether they are one- or two-sided<br>*Only common tests should be described solely by name; describe more complex techniques in the Methods section.*
☐	☒	A description of all covariates tested
☐	☒	A description of any assumptions or corrections, such as tests of normality and adjustment for multiple comparisons
☐	☒	A full description of the statistical parameters including central tendency (e.g. means) or other basic estimates (e.g. regression coefficient) AND variation (e.g. standard deviation) or associated estimates of uncertainty (e.g. confidence intervals)
☐	☒	For null hypothesis testing, the test statistic (e.g. *F*, *t*, *r*) with confidence intervals, effect sizes, degrees of freedom and *P* value noted<br>*Give P values as exact values whenever suitable.*
☐	☒	For Bayesian analysis, information on the choice of priors and Markov chain Monte Carlo settings
☒	☐	For hierarchical and complex designs, identification of the appropriate level for tests and full reporting of outcomes
☐	☒	Estimates of effect sizes (e.g. Cohen's *d*, Pearson's *r*), indicating how they were calculated

Our web collection on statistics for biologists contains articles on many of the points above.

Software and code

Policy information about availability of computer code

Data collection	Otolith microchemistry data was collected as blank subtracted count data for each ablated pit in MassHunter Software (Agilent Technologies) and converted to element concentrations (ppm) by manual calculation using Ca as the internal standard.
Data analysis	All statistical and GIS analyses were conducted in R statistical software version 4.0.2. The packages, "nnet" and "mgcv" were also used. Data analysis and modelling R code developed here is available in a public GitHub repository- https://github.com/NeilMBurns/Element_Isoscape_geolocation20 (http://doi.org/10.5281/zenodo.4088644)

For manuscripts utilizing custom algorithms or software that are central to the research but not yet described in published literature, software must be made available to editors and reviewers. We strongly encourage code deposition in a community repository (e.g. GitHub). See the Nature Research guidelines for submitting code & software for further information.

Data

Policy information about availability of data

All manuscripts must include a data availability statement. This statement should provide the following information, where applicable:

- Accession codes, unique identifiers, or web links for publicly available datasets
- A list of figures that have associated raw data
- A description of any restrictions on data availability

The data supporting the findings presented here are available online from the University of Glasgow Enlighten database - https://doi.org/10.5525/gla.researchdata.1040

Field-specific reporting

Please select the one below that is the best fit for your research. If you are not sure, read the appropriate sections before making your selection.

☐ Life sciences ☐ Behavioural & social sciences ☒ Ecological, evolutionary & environmental sciences

For a reference copy of the document with all sections, see nature.com/documents/nr-reporting-summary-flat.pdf

Ecological, evolutionary & environmental sciences study design

All studies must disclose on these points even when the disclosure is negative.

Study description	Fish were collected by bottom trawl from seven sample locations in October 2014 and ten sample locations in March 2015 from across the west coast of the UK. 200 individuals were collected from each sample site (n=100 > 20 cm and n=100 < 20 cm) and 299 otoliths were used in microchemistry analysis.
Research sample	Whiting (Merlangius merlangus) total Length (TL, to nearest mm) was recorded, sex was determined during dissection and both sagittal otoliths removed using ceramic tipped forceps. A TL stratified random sample (38%) of left sagittal otoliths was used for annual age estimation. The right was used for LA-ICP-MS microchemistry analysis. The 2014 cohort was identified from annual increment counts and otoliths from age-0 (caught in October 2014) and age-1 (caught in March 2015) were used in the analysis. This species was selected with the aim of understanding its relative success compared to other gadoids which occupy these waters E.g. cod & haddock).
Sampling strategy	Sample sizes were based on previous work examining otolith microchemistry. In the current study, sample group sizes at each location exceeded the numbers successfully used in previous published works.
Data collection	Fish sample collection was carried out by crews of the Scottish West Coast Groundfish Surveys (SCOCGFS) and Northern Ireland Ground Fish Survey (NIGFS). Fish dissection, data recording and otolith processing was conducted by N. Burns (author). LA-ICP-MS sample analysis was conducted by Kirsty Donald and Craig Robinson and assisted by N. Burns at Marine Scotland.
Timing and spatial scale	Samples were collected at October 2014 and in March 2015. These time points correspond to post-settlement and the start of spawning for whiting and correspond to the 2014 cohort. The spatial scale of the current work was selected to examine two fisheries stock assessment units to the west of the UK for this species.
Data exclusions	10 sampled otoliths, 6 from the October 2014 sampling time point and 4 from March 2015, were excluded from the analysis. These samples were excluded at the point of visual examination because of damage caused during preparation of the otolith material.
Reproducibility	No repetition was conducted although repeated LA-ICP-MS runs over several days produced comparable results from different randomly ordered otolith samples. The results are also in-line with those expected from other published otolith microchemistry studies conducted to the west of the UK
Randomization	Fish samples were stratified by length to ensure sufficient numbers for age estimation. Otolith selection for LA-ICP-MS analysis was conducted at random and the otoliths were processed in random order to prevent sample batch bias. All random numbers were generated in R 3.6.1 using the runif function.
Blinding	Blinding of sample origins was not possible at the point of fish dissection because of the spatial nature of the study. However, from this point forward in the sample processing strategy otolith samples were allocated an ID number and randomly ordered to prevent bias.

Did the study involve field work? ☒ Yes ☐ No

Field work, collection and transport

Field conditions	Sample collection was carried out by crews of the Scottish West Coast Groundfish Surveys (SCOCGFS) and Northern Ireland Ground Fish Survey (NIGFS) onboard research vessels.
Location	The scientific trawl surveys supplying the fish samples were conducted at seven locations in October 2014 (-3.756, 54.400; -6.486, 56.726; -7.233, 58.283; -5.408, 53.581; -5.282, 55.091; -4.815, 55.463; -6.834, 57.730) and ten locations in March 2015 (-3.711, 54.273; -7.352, 58.262; -6.704, 57.129; -5.223, 55.414; -6.761, 56.188; -5.419, 58.673; -3.763, 53.482; -8.683, 57.309; -5.690, 53.626; -5.890, 58.005). The longitude and latitudes displayed are for the mid point of the trawls. Trawls were conducted at depths between 30 m and 200 m.
Access & import/export	All samples were collected as part of the normal operating procedures for the Scottish West Coast Groundfish Surveys (SCOCGFS) and Northern Ireland Ground Fish Survey (NIGFS). All were sourced from within the UK EEZ. Fish were transported dead and frozen.
Disturbance	The current study used fish which were collected during the normal activities of the Scottish West Coast Groundfish Surveys (SCOCGFS) and Northern Ireland Ground Fish Surveys (NIGFS). No additional trawling was required.

Reporting for specific materials, systems and methods

We require information from authors about some types of materials, experimental systems and methods used in many studies. Here, indicate whether each material, system or method listed is relevant to your study. If you are not sure if a list item applies to your research, read the appropriate section before selecting a response.

Materials & experimental systems

n/a	Involved in the study
☒	☐ Antibodies
☒	☐ Eukaryotic cell lines
☒	☐ Palaeontology and archaeology
☐	☒ Animals and other organisms
☒	☐ Human research participants
☒	☐ Clinical data
☒	☐ Dual use research of concern

Methods

n/a	Involved in the study
☒	☐ ChIP-seq
☒	☐ Flow cytometry
☒	☐ MRI-based neuroimaging

Animals and other organisms

Policy information about studies involving animals; ARRIVE guidelines recommended for reporting animal research

Laboratory animals	The study did not involve lab animals.
Wild animals	Whiting (Merlangius merlangus) were caught as part of the normal activity of scheduled scientific trawls. Fish were killed on deck,bagged and frozen for transport.
Field-collected samples	All samples were killed on deck before transport to the lab.
Ethics oversight	No ethical approval was required as samples were acquired as a result of normal scientific fisheries survey operations.

Note that full information on the approval of the study protocol must also be provided in the manuscript.
